# Few opportunities to influence decisions regarding the care and treatment of an older hospitalized family member: a qualitative study among family members

**DOI:** 10.1186/s12913-017-2563-y

**Published:** 2017-08-31

**Authors:** Ingrid Nyborg, Lars Johan Danbolt, Marit Kirkevold

**Affiliations:** 1 Institute of Health and Society, University of Oslo, Blindern, P.O. Box 1130, NO-0318 Oslo, Norway; 2 Innlandet Hospital Trust, Kyrre Grepps gate 11, NO-2819 Gjøvik, Norway; 3 Norwegian School of Theology, Majorstuen, P.O. Box 5144, NO-0302 Oslo, Norway; 4 Director of The Center for the Psychology of Religion, Innlandet Hospital Trust, P.O. Box 68, NO-2312 Ottestad, Norway

**Keywords:** Adult, Aged, Aged, 80 and over, Middle age, Decision making, Family, Hospitals, Older people, Patient participation, Qualitative research

## Abstract

**Background:**

The drive towards patient involvement in health services has been increasingly promoted. The World Health Organisation emphasizes the family’s perspective in comprehensive care. Internationally there is an increased emphasis on what patients and their family tell about the hospital experiences. However, current literature does not adequately address the question of participation experiences among relatives of older hospitalized family members. There is a paucity of research with a generational perspective on relatives’ opportunities to exert influence. The aim of the study was to explore relatives’ experiences of opportunities to participate in decisions about the care and treatment of older hospitalized family members and whether there are different experiences of influence to the relatives’ age.

**Methods:**

This was an explorative study applying individual qualitative interviews. The interviews were analysed following hermeneutic methodological principles. Two Norwegian geriatric wards participated: one at a university hospital and one at a local hospital. Twelve participants, six women and six men, were purposively selected. The relatives were aged from 36 to 88 (mean age 62) and were spouses, children and/or children-in-law of patients.

**Results:**

The relatives’ experienced opportunities to exert influence were distributed along a continuum ranging from older relatives being reactive waiting for an initiative from health professionals, to younger adults being proactive securing influence. Older “invisible” carers appeared to go unnoticed by the health professionals, establishing few opportunities to influence decisions. The middle-aged relatives also experienced limited influence, but participated when the hospital needed it. However, limited participation seemed to have less impact on their lives than in the older relatives. Middle-aged relatives and younger adults identified strategies in which visibility was the key to increasing the odds of gaining participation. The exceptional case seemed to be some older carers’ experiences of influencing decisions with the help of professionals.

**Conclusions:**

Our findings suggest that experiences of influence were limited regardless of age. However, the results indicated that participation among relatives decrease with age while vulnerability for not having influence seemed to increase with age. The problem of patient choice most clearly manifested among the older carers, which might indicate that the relatives’ age sets terms for opportunities to participate.

## Background

Patients are entitled to participate in decisions regarding their care and treatment, and have the right to receive the information necessary to obtain insight into their health condition and the content of the health care provided. The family may gain influence and participation in the process of care and treatment if the patient gives his or her consent [1, 2].

This study concerns relatives’ experiences of opportunities to participate in decisions about the care and treatment of older hospitalized family member and whether there are different experiences of influence related to the relatives’ age.

In this study, we understand participation to mean the “involvement in the decision-making process in matters pertaining to health” ([3, 4] (MeSH-term)), and decision making as “the process of making a selective intellectual judgment when presented with several complex alternatives consisting of several variables, and usually defining a course of action or an idea” [3, 5, 6]. Furthermore, we understand influence on decision making to be a phenomenon that varies in extent and context in line with Thompson (2007). Thompson (2007) described different levels of patient involvement and participation ranging from non-involvement, seeking and receiving information, information-giving, possibly dialogue, shared decision making and autonomous decision-making [7]. Each level depicts the “patients’ relative power to influence decisions” ([7] p. 1302). Achieving a particular level in one situation does not automatically predict a move to the next level. The level of participation is, at any given time and whatever the personal preferences, depending on health professionals, settings or illness [7]. In this study, the terms influence refers to the capacity or power of relatives, by direct or indirect means, to impact on the decisions-making processes about care and treatment of their older hospitalized relative [7].

There is a paucity of research examining family experiences in hospital, and research indicates that relatives’ influence on health services generally is limited [8–12]. A recent study found that whether the relatives were next-of-kin to a spouse, a child, an adult child, a parent or a sibling, and whether the diagnosis was somatic or psychiatric, the experiences with health services seemed to be similar. They reported a lack of information, inclusion and collaboration in the care of their ill family member [8].

With respect to the relatives of older hospitalized family members, studies examining family experiences in hospital have mostly treated the relatives as a homogenous group [9, 10, 13–15]. A qualitative study on expectations, communication and care decisions among families and caregivers of older people, uncovered differences between older and adult relatives [16]. Some of the older relatives had health or cognitive problems impacting on their ability to provide care for another. The adult relatives had concerns about their other responsibilities, such as family and work. Regardless of age, being a relative of a patient in a geriatric hospital ward was stressful. The major themes emerging from the interviews centred on the family caregivers’ need for consistent reliable communication and involvement in care decisions [16].

Regarding exchange of information, responsibility for the patient’s wellbeing in hospital and for the patient’s compliance with the daily regimen, Norlyk (2012) suggested that relatives were the ‘extended arms’ of health professionals [17]. According to other studies on user participation among older patients, the relatives were, by patients, perceived to be ‘the extended arms’ of themselves; they delegated decision-making to relatives [18, 19]. Some present research emphasizes the relatives’ contribution to the support and enhancement of the level of patient participation [20, 21]. A review of the evidence on hospital discharge planning for frail older people and their family, indicated that family participation could improve the discharge process [22]. The study on informal caregivers’ participation when older adults in Norway are discharged from the hospital, found that the younger relatives (mean age 55) experienced a higher degree of involvement in receiving and providing information to hospital staff than did the older (mean age 80) [23]. At hospital, the younger, but significantly less the older, relatives reported receiving sufficient information about the patient’s medical conditions, and the younger experienced to a higher degree than the older, that the patient was sufficiently informed. The study suggested that older patients assisted by older relatives, might be exposed to higher risk of inadequate participation needed for an appropriate discharge to home [23]. Furthermore, the study found that the younger generations of carers seemed to have better chances for exerting influence on decisions related to the care and treatment of their older relative, and that for the younger relatives it was imperative to gain influence on decisions in matters that affected their own life [23].

This study is a part of a larger research project focusing on user participation among older hospitalized patients and their relatives. The first study found that older patients addressed their difficulties of participating by authorizing family members to act and participate on their behalf [18]. The second study compared and contrasted older patients’ and their relatives’ experiences of participation in decision-making processes regarding the planning of everyday life after discharge from hospital [24]. Participation in making decisions appeared to be random and limited for both patients and their relatives, and conflicting for the families as a whole. The decision-making processes seemed to be limited to the hospital context; decisions appeared to be settled without considering the patient’s broader life context in which family played a role. The relatives told they provided assistance to the patients on a daily basis, but were side-lined even if the decisions made at the hospital affected their everyday life [24]. These results are consistent with previous research [5, 23, 25–27]. The results from study two seemed to suggest a pattern of age-related differences; the relatives’ influence and participation seemed to decrease with age while vulnerability for not having influence seemed to increase with age. Limited participation in decisions seemed to affect older carers’ lives more than the middle-aged relatives. However, this was not explored systematically in that study. Consequently, the next step in the project was to analyse this issue in-depth. That is the topic of this paper.

### Aim

The aim of the study was to explore relatives’ experiences of opportunities to participate in decisions about the care and treatment of older hospitalized family members and analyse whether there are different experiences of influence related to the relatives’ age.

## Methods

### Design

The study had an explorative design and was informed by Brinkmann and Kvale (2015) and the phenomenological hermeneutical method for researching lived experience developed by Lindseth and Norberg (2004) [28, 29]. According to the latter, the most basic way to gain access of human experiences is to listen to others’ stories about the way they act in various situations. Experience is implicit in a situation and in the story about the situation. Humans organize experiences so that they answer questions like: ‘what’, ‘why’, ‘who’, ‘how’, ‘with whom’, ‘to whom’ and ‘for whom’ [28]. The study complied with the Consolidated Criteria for Reporting Qualitative Research (COREQ) [30].

### Setting and participants

The empirical part of this study was conducted in 2013 in two Norwegian geriatric wards, one at a university hospital and one at a local hospital. The wards offered a treatment and rehabilitation program including patients aged 65 and over, with multi-morbid conditions and complex health problems. In this study, the typical reason for hospitalization was acute functional decline, fall or inadequate intake of fluid and food.

The inclusion criterion in the study included being a Norwegian speaking relative of a patient admitted to one of the two geriatric wards. We applied a purposive recruitment strategy to achieve maximum variation of the sample. The head nurses gave geriatric nurses the authority to recruit relatives by a face-to-face approach when the relatives visited the wards, or by telephoning relatives the nurses had met in the wards. The nurses were asked to recruit relatives with different relationships to the patient, gender and age, as we assumed that these characteristics might impact on the opportunities to participate in decision-making. As most patients in the wards were 70 years and above, available spouses and children were of a certain age. The classification of age complies with the MeSH (Medical Subject Headings) terms [3] (see Table 1). In this study, the term *older* refers to the participants aged 65 and over, *middle-aged* to participants between 45and 74 and *younger adults* for participants less than 45 years.

**Table 1:** Participants

Relatives			Patient
Relation to patient	Age of relatives	Work status	Age of patient
Wife	Older	Retired	Older
Husband	Older	Retired	Older
Wife	Older	Retired	Older
Daughter	Older	Retired	Older
Son-in-law	Older	Retired	
Daughter	Middle Age	Retired	Older
Son	Middle Age	Employed	Older
Daughter	Middle Age	Employed	Older
Son	Middle Age	Employed	Older
Daughter-in-law	Middle Age	Employed	
Son	Middle Age	Employed	Older
Son	Younger Adult	Employed	Middle Age

Approximately 25 potential study participants were assessed and invited to join in the study. Nine relatives declined the invitation due to time pressure or of reasons we do not know; sixteen relatives were enrolled in the study. The geriatric nurses provided written and oral information to potential participants, who were given time to consider participation in the study. Written consent was obtained and assurances of confidentiality and anonymity were given. One relative declined to participate. One relative dropped out because of stress and time pressure, one relative never had time for an interview appointment, and one relative dropped out because the patient became sicker. Accordingly, twelve relatives participated in this study. There was no relationship between the interviewers and the potential participants prior to study commencement.

Four participants lived in urban communities and eight lived in rural communities. Six relatives were retired from work. Six relatives were employed (see Table 1). Among the participants were men and women with professions related to health services and who had insights into specialised rehabilitation services and deep knowledge of hospitals. They were also well informed about user and patient rights. Other participants had technical practical or administrative occupations.

Table 1 show that five participants were older, six participants were middle-aged, and one was a younger adult. The participants consisted of six women and six men.

### Data collection

Individual interviews were used to collect data. The purpose was to obtain in-depth information about relatives’ experiences of participation, and was conducted by the first author in 2013. The first author, who is female, was a fulltime PhD candidate at the time of the study. She had leave of absence from work as an occupational therapist in a geriatric ward. This background might have impacted on the data collection by influencing what caught her attention in the interview situations (e.g. regarding how life circumstances might have an impact on the individual’s possibilities of gaining user participation and how health services adapted user participation).

An interview guide aimed to uncover experiences of user participation was developed based on key documents [31–33]. The interview guide is summarized in Table 2.

**Table 2:** Interview guide

Themes	
Being the relative of an older hospitalized family member Participation and influence	How are you related to the patient? Can you tell me how the patient was hospitalized and what happens when you are present? What is your situation? In your opinion, what is it important that you tell the staff and the nurses about yourself and your situation? Can you share some of your thoughts about how you have been welcomed as a relative? Are you being asked about your own experiences and wishes when it comes to the specific situation of your ill relative and its impact on your situation? What do you consider important to you and your situation? Can you tell me about your needs/wishes?

Duration of the interviews ranged from 19 to 81 min with an average of 35 min. An audio recorder was used. The interviews were conducted at the preferred location of the relatives: four at the hospital, three at home, two at the relatives’ workplace, and one at a near-home location. Ten relatives were interviewed while their family member stayed at hospital, or within a few days after the patient’s discharge from hospital. Two relatives were interviewed respectively 11 days and nearly three months after the patients stay at hospital. The reason for this was time pressure on the part of the relatives.

The number of relatives to be interviewed was not predetermined. The recruitment process ended when experiences of participation kept recurring in the interviews.

The interview guide was not pilot tested. The first author was trained by the supervisors, and a nurse trained in the craft of research interviewing was present and participating in some of the early interviews. After the interviews, the trained interviewer gave feedback on interview performance and critically discussed possible interpretations of the relatives’ accounts. The subsequent interviews were more conversation like with the interview guide used as a check list to ensure addressing all relevant topics. Discussions between the supervisors and the first author were described and reflected on in memos [30], which were written immediately after each interview, and were later subjected to critical reflections by the research group. The memos provided additional information about the interview situation, interaction, emotional expressions and the relatives’ accounts. All interviewers were women.

The interviews were transcribed verbatim by the first author, including nonverbal audible signals such as laughter, sighs and pauses helping the researchers to comprehend the interviews within their particular context [30]. Researcher triangulation of data enhanced the credibility of the interpretation. The supervisors had different professional backgrounds and research experiences, which ensured a diversity of perspectives. Over time the authors critically discussed and reflected on the interpretations and broader perspectives and possible meanings were uncovered. Summarizing, to enhance the trustworthiness we have attended to the integrity of data, the balance between reflexivity and subjectivity (as bias enters as soon as a research question is asked in a particular way), and we have sought to provide a transparent account of all aspects of the research process [30, 34].

### Data analysis

This study applied a phenomenological-hermeneutic analytic approach [28, 29]. The main tool to manage the interview texts was Microsoft word. The initial reading of the transcribed data aimed to gain a preliminary understanding of the phenomenon (experiences of influence and participation) and its context [29]. The next reading was to create an initial structure of meaning units, themes and subthemes in order to clarify the significant meanings in the texts. First, the text was divided into meaning units, i.e. shorter or longer parts of the text related to the research question. These were condensed into brief everyday words capturing the essential meanings. Condensed meaning units that were similar were then abstracted to form sub-themes, which were next assembled and abstracted into themes [28]. Table 3 show an illustration of the analytic process from interview text to themes via meaning units, subthemes and themes.

**Table 3:** Illustration of the analytic process

Meaning units	Condensed text	Sub-themes	Themes
I was visiting my spouse every day, but did not attend the ward round. The nurses and the others were so nice, but I think that some information [from me about my husband/wife] would have been for the better; they didn’t ask me about anything. (Older relative) If I receive all the help I am offered at home, I might as well move out myself. The last time the ambulance was here, I asked them how sick you would have to be to get into a nursing home. Someone needs to do something, try to apply for a place! (Older relative)	Wanting to influence decisions but being reactive in that respect. Taking little or no initiative to exert influence.	Waiting for professionals to initiate contact	Neither seen nor heard
I have phoned to all kinds of healthcare services. For thirteen years! No one has called me. Mom wants to live at home, something she has told everyone in the systems, you know. So, therefore, it has been easier for them to send her back home, of course. From where it could be, the hospital or others services. (Older relative)	Making a huge effort into getting adequate help from healthcare services to the patient	Fighting to be heard	Unwilling acceptance

We understand hermeneutic analysis to be an active and reflexive approach to theme development. Each interview text was given equal attention in the analytic process; the interpretations were validated by re-reading the whole text several times in light of the meaning units, subthemes and themes and the other way around.

At some point an age-specific dimension emerged; participants seemed to describe different experiences depending on age. We searched for other patterns as well, e.g. the kind of family relation between relative and patient, gender, relatives being health professionals, being able to take leave of absence from work, and/or having the opportunity to be present in the hospital where user participation materialised. From our analysis, we concluded that all these aspects seemed important for the experiences of participation in decision-making processes. However, they played out differently depending on the age of the relatives, which we have tried to capture in the findings.

**Fig. 1:**
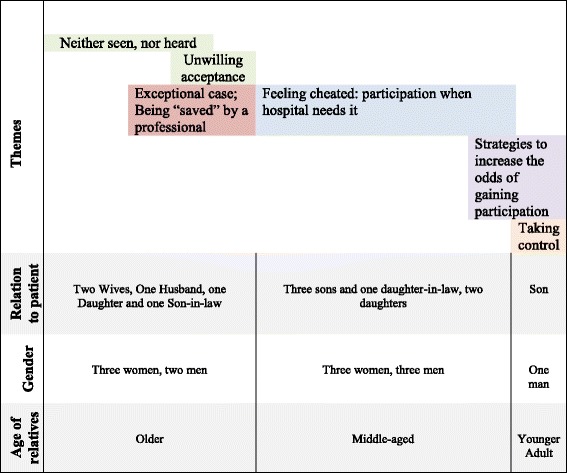
From invisible and reactive to visible and proactive: a continuum of influence on the care and treatment of older family member

Two patterns stood out in the texts because of the extremely different experiences of participation in decisions about the care and treatment of the hospitalized family member. Whereas the experiences recounted by older relatives reflected an invisible and reactive attitude to participation, the younger adult’s experiences reflected a visible and proactive attitude to participation. By a hermeneutic turn in the analysis, these two extremely different patterns became pivots to the continued analysis, and the whole text was re-read in light of these identified patterns. This resulted in a continuum of opportunities for relatives to exert influence on care and treatment of older family members (Fig. 1).

### Ethical considerations

The study was approved by the Regional Committee for Medical and Health Research Ethics South East Ref. 2012/1598. To protect participants’ anonymity, two hospitals were included in the study. It was emphasized that participation in the study was voluntary and that consent could be withdrawn at any time and without any kind of repercussion.

## Results

All relatives appeared to experience the opportunity to influence decisions about the care and treatment of an older family member to be dependent on permission from the patient and/or the health professionals. Being a relative with a health professional background appeared to make no difference concerning the relative power to influence decisions. Apart from the younger adult, who was a man, the age groups were equally distributed in terms of gender. The variety of family relationships between the relative and the patient were equally distributed among the older and the middle-aged groups (see Fig. 1). Thus, we interpreted the different experiences of opportunities to participate in decisions about the care and treatment of the patient to be strongly related to the age of the relatives.

The relatives’ experiences of influencing decisions appeared to form a continuum ranging from having scarcely any such experience to report, to experiences of taking control. Their opportunities to exert influence ranged from being “invisible and reactive” (i.e. waiting for an initiative from the health professionals) to being “visible and proactive” (i.e. securing influence). The sliding yet overlapping transitions between the different experiences of influence may be illustrated as in Fig. 1.

Figure 1 show the continuum of experienced opportunities to participate in decisions about the care and treatment of older family members, and puts this continuum into the contexts of relationship, gender and age.

Relatives communicated how time consuming it was to be relatives. Some of the relatives who were employed pointed out the necessity of being able to take leave of absence from work, or taking holiday time at the time their parent was admitted to and discharged from hospital. On a daily basis, the older carers played a large part in the care of the older family member, but experienced limited opportunities to influence decisions *affecting* their daily life. Whether the older carers were present at the hospital or not, they appeared to be “invisible” and go unnoticed by the health professionals. This indicates that taking initiative seemed to be a prerequisite even to get possibilities to partake. According to a middle-aged relative and the younger adult, visibility and presence were key strategies to increase the odds of gaining participation. The younger adult experienced participation by taking control himself. Those of the older relatives who did experience some influence seemed to be exceptional in the sense that a particular professional they encountered discovered and verbalised their needs and took action accordingly. In the following sections, the findings are described in further detail.

### Neither seen nor heard

The experiences recounted by the two oldest women referred to their own invisibility as well as that of the health professionals. The women led their “hidden” lives behind the four walls of their own home adapting to their spouses’ needs. The wives said they had limited access to transportation, and that they rarely contacted the healthcare services. When the health providers contacted the wives, this was generally by telephone to give information (about decisions made by the professionals), or ask for information related to the health of the patient. It therefore seemed they were dealing with faceless and nameless professionals who they referred to as “they” and “them”. The wives appeared unclear whether the callers had the authority to make decisions, and if so, what about. Once, one of the wives made a phone call herself but her voice was not heard:

*“When my husband last returned home from the hospital, I rang the community nurses and asked if I could get some help. Well, that would mean we would have to employ more people, she said! But could you please come and put on the pain plaster? So they came around twice, and then they asked me if I could do it. That was all. I have never asked for anything else after that. Perhaps they ought to think about the person who isn’t sick as well as the person who is”* (An older relative).

The wives asked the interviewer to help them exert influence over the healthcare services (to extend a stay in a nursing home). They asked how ill their spouses would have to be to warrant a place in a nursing home:

*“How sick would you have to be to get into a nursing home? How far can you push yourself?! I don’t think I can take much more!”*

In contrast to the older wives, an older husband told that he was present at the hospital almost every day. Nevertheless, he did not feel to be seen and heard; neither the health professionals nor his wife gave him opportunities of any kind, to participate in making decisions:

*“Yes, the discharge came as a surprise. Nobody told me anything. My wife was far from healthy. I was worried. But our son is ever so kind, and moved out of his bedroom. If they had been extremely busy at the hospital, I wouldn’t have mentioned it, but there were lots of empty beds”.*

### Unwilling acceptance

Two of the older relatives, who were over 70 years of age at the time of the interview, and the younger adult, explicitly discussed the challenges related to patient choice. They described experiences of the patient appearing to failing not recognize the carers’ and the families’ situation when making decisions. For the older relatives, providing care involved maintaining three households: their own home, the childhood home and the mother’s flat. The relatives explained that they had little control of their own situation and no influence on the patient’s decisions. They talked about a 60-year career as carers and described how they had fought for the patient to receive appropriate care, but the system had always supported her mother, whose preference was generally to refuse such care. The patient had been hospitalized two years earlier and the situation had now become equally precarious. The relatives said they had contacted a number of service providers, and the general practitioner, to ask if someone could find an emergency nursing home bed, which reputedly was impossible. The following day a temporary employee from the community nursing service managed to get her mother admitted to hospital:

*“Is there no-one who can override THE PATIENT? The doctor attended to my mother on the Thursday, and she was so poorly! Then I talk to the doctor on the following Friday and she says ‘we cannot hospitalise a patient when she herself says no’. Says the doctor. And then a TEMPORARY employee from the community nursing service gets hold of an ambulance and sends my mother to hospital. I thought that was brilliantly well done.”* (An older relative)

### Exceptional experiences; being “saved” by a professional

Despite a general experience among the older relatives of not being seen or heard, two of the relatives recounted exceptional experiences of be “saved” by professionals who saw their struggles, verbalised their needs and took action accordingly. The older relatives themselves related their lack of ability to take care of their own needs to their old age. They told that they had difficulties asking for help and was grateful to nurses who saw their needs and acted on them. According to older relatives, self-sacrifice is a particular characteristic of their generation; they felt that things were different for younger people. An older relative felt unable to verbalise her own needs vis-à-vis the health professionals, and was even less capable of influence decisions in a way that might improve her own life, but would go against her husband’s wishes:

*“It feels bad to talk about my husband in this way, but … I had better use the words of the nurse in the hospital. She said that if you agree with me, she said, I would recommend that you apply for a long-term place for your husband straight away. For there is no sense in you wearing yourself out. Said the nurse. Talking like that about your husband, feels a bit, you know, you sort of feel that you need to try your very best, for as long as possible. But of course, once it starts wearing you down, it just gets too much.”* (An older relative)

To look after the most elderly members of the family was, according to older relatives, an obligation for people of their generation, even if this was at the expense of their own lives. Some compared being responsible for the patient to having a child at nursery school, and said they had handed over a whole book to the hospital about the patient’s condition. These relatives told that a nurse had confirmed that their situation was intolerable and had virtually demanded that the primary healthcare service find a nursing home place. The older relative expressed: “The nurse took responsibility. She addressed the problem. I was deeply pleased.”

### Feeling cheated: Participation only when the hospital needs it

Generally, the middle-aged relatives felt that no significant influence had been obtained. In an attempt to influence the care of the older hospitalized relative, they collaborated with several family members, who gathered information when visiting the patient. The following statement was representative of the middle-aged relatives and their toleration of their own limited participation in relevant decisions:

*“It all depends a lot on your health, yes, it all depends on your current situation. Had I had a lot to cope with personally, poor health and that sort of thing, it may well have been more difficult for me to take on this role. As it was, I didn’t even reflect on it. I feel I have the competence required of a next-of-kin, and I don’t consider it a burden. But how it will feel in 30 years, I don’t know, really. It is difficult to tell.”* (A middle-aged relative)

All middle-aged relatives told they had assisted the hospital by providing information about the patient. However, they were frustrated by the absence of follow-up dialogues. Regarding other responsibilities such as family and work, the middle-aged relatives called for opportunities to influence decisions about practical tasks. A relative told that the providing of information had cost her a great deal; she did not want “to tell on dad”. However, on the hospital’s request, she had given information about the patient’s health and level of functioning at home. In return for providing information she wanted dialogue with the professionals, but no dialogue was initiated. On the contrary, referring to the patient being angry and stressed on the ward, a nurse called and asked the relatives to arrange for a short leave from the hospital:

“*With regard to the leave, the hospital collaborated with us, on their initiative. But when telling them everything about dad’s behaviour at home, I felt somewhat cheated when I received nothing in return*.” (A middle-aged relative)

### Strategies to increase the odds of gaining participation

To get in position for participation, a middle-aged relative and the younger adult recounted employing different strategies. In order to boost user participation, they strategically develop interpersonal relationships with the professionals and earned goodwill and acceptance by providing personal care for the patient. Furthermore, using clear communication, e.g. presenting an unambiguous message to the professionals, and preparing themselves by reading white papers, legislation and research posted on the internet were strategies employed. This was something the older relatives did not experience to master: “*We don’t have a computer, so we’re part of a generation that’s becoming extinct, I believe*”.

The singularly most important strategy was to be visible and present in the hospital in order to receive and provide information: knocking on the door of the ward office, requesting conversations with the nurse in charge and taking part when doctors were doing their rounds.

*“It’s all-important [to be present at the hospital] to catch what is going on. You never receive any information, there is no telephone contact, but because I have been here a lot, you get to know what you need to know. But you have to ask. So I listen out all the time.” (*A middle-aged relative)

### Taking control

The younger adult experienced that the patient, at first, excluded him from participation in decisions about the care and treatment, but the health professionals carefully did listen to him. The younger adult reported that he exerted influence by taking control and organising meetings attended by the professionals, the patient and the relative, and felt that he in this way (“no tricks, just common sense”) helped the patient to make the right decisions.

During the interview, the younger adult’s main concern was how to organise a conversation that would allow the patient to make good choices, and he discussed the problem of patient choice. The son felt that the prevailing logic of choice was counterproductive, not only restricting the relatives’ level of participation but also the patient’s level of involvement, arguing as follows: the son brought his mother to the general practitioner who did not give his mother healthcare assistance because his mother had not chosen this for herself. This, according to the son, indicated that the doctor considered it to be more important to give the patient choice than to involve the patient and relative in conversations about best interests of the patient. The younger adult was of the opinion that doctors associated making choices with participating in decisions as if they were the same phenomenon. By focusing on treatment choices, the patient’s opportunity to exert influence on a singular decision increased, but the patient’s participation in a process of making decisions involving several considerations, decreased:

*“Even though you should never interrupt a senior consultant while talking, I had to tell her that she would be better off talking about my mother’s medical condition. The doctor came up with a number of different treatment alternatives, and the many options confused my mother. The doctor started the wrong way around. At any rate, it did not make for a good situation.”* (The younger adult)

The younger adult questioned whether the focus on individual choice in user participation ideology did in fact compromise the ethical principles of patient care. He argued that when a patient was as ambivalent as his mother, who was making choices that potentially would endanger her own life, rather than the good decisions, then this was a moral problem. According to the son’s reasoning, giving his mother, in her present situation, a number of options, could result in his mother making decisions that was contrary to her own wishes. In his opinion, participation by patients and their relatives would need to take place in a forum in which patients, the relatives and health professionals openly discuss the best interests of the patient.

## Discussion

The aim of this study was to explore relatives’ experiences of opportunities to participate in decisions about the care and treatment of older hospitalized family members and analyse whether there are different experiences of influence related to the relatives’ age. We found that most of the relatives experienced low levels of user participation regardless of age. This is in line with existing literature [5, 8–12, 23, 25–27, 35], but contrary to the relatives’ preferences of participation. Nevertheless, age did seem to impact on the relatives’ opportunities to influence decisions. In the following we discuss these findings and their implications.

The older relatives in this study adopted a reactive approach to participation. In the interviews, they expressed intentions and wishes to influence decisions, but in a responsive and reserved way. The reactive attitude exposed by older relatives might challenge the current participation ideology which is based on individualism and requires proactive partners in health care [4, 36]. The older generation’s commonly held values of solidarity and community might conflict with such ideas [36]. The first study in the larger research project of which this paper is a part, highlighted the ambiguous participation on the part of older hospitalized patients. They seemed to gain influence through active and passive approaches [18] in line with previous research [36]. When older patients experienced difficulties in participating in decisions regarding treatment and care, they delegated decision making to the relatives and the professionals [18]. This kind of active and passive approaches to gain influence might be common features of older people, both relatives and patients.

In our results, patient choice emerged as a possible *problem.* Some relatives experienced that choices made by patients (and professionals) seemed to disregard the family’s needs and life circumstances, and imply care resources the relatives did not possess. Although the current participation ideology based on liberalism emphasises individuals’ free choice [36–38], the results might indicate that the individual patient, and not the individual relative, had opportunities to make choices for themselves. Cash et al. (2013) claimed that the ideal of individual choice remains largely absent from policies directed at informal caregivers, and that research has been limited in developing an understanding of the underlying choices, or lack thereof, in providing informal care [38]. The emergence of liberalism within welfare policy has, according to Cash et al. (2013), created an inequity for older carers, who are not offered the same degree of choice as other older people, e.g. older patients. Due to factors such as age and relationship to the care receiver, the problem of patient choice is particularly the case with spousal care [38], which our study might underpin.

The younger adult questioned whether the focus on choice in user participation ideology did in fact compromise the ethical principles of patient care. Mol (2008) differentiated between the logic of care and the problem of patient choice with the same arguments [37]. The logic of care does not construct patients as passive: “they do not primarily figure as subjects of choice, but as the subjects of all kinds of activities” ([37] p. 8). This logic recognizes that patients can’t be separated from family, friends and other support systems [37]. Our results indicate that choices made at hospital had unintended consequences, and that the problem of patient choice most clearly affected the older relatives who did not manage the care responsibility assigned to them.

On the subject of making choices in matters of their own concerns, the middle-aged relatives called for opportunities to influence decisions about practical tasks in order to coordinate care with other family responsibilities. Relatives have no autonomous right to participate in decisions about care and treatment of adult family members unless on behalf of the patient [39]. However, an approach to participation by relatives of older patients, that is not merely an extension of patient participation, has been suggested by an integrative literature review on carer engagement in the hospital care [14]. The review argued for establishing an integrated model of carer engagement whereto the relatives can participate, e.g. through information sharing, shared decision-making, carer support and education, and communication with the health professionals [14]. In line with a study focusing on relatives as competent collaborative partners [11], relatives in our study might be interpreted as such. However, the middle-aged relatives seemed to cope with experiences of low participation better than the older people, possibly because they could handle the consequences for themselves due to better health and help from other family members.

We uncovered a few exceptional experiences of user participation, in which participation was facilitated by an attentive professional who discovered the relative’s need for assistance. Previous studies have reported that that the nurses’ attitudes and how they approach the family are the strongest predictors for collaboration to happen [35, 40]. Valuing relatives has been shown to open up possibilities to influence decisions [35, 40]. Furthermore, “active listeners” among the staff promote family participation in the care of older patients in institutional settings [41]. This was evident in this study as well, which underscores the importance of professionals facilitating user participation among relatives.

A recent study proposed that older patients in the emergency department should be treated as a specialty population in the sense that this group is a vulnerable population and should be placed in age-friendly environments and being met by specialised staff members [9]. Our study proposes that relatives of frail older patients in general, but particularly older vulnerable relatives should be treated as a “specialty population” in the hospital. By this we mean that they need special attention and involvement in decisions regarding the treatment and care of their hospitalised relative. Health professionals should be particularly aware of older relatives who need help to express their own needs for support [23].

A current review of available knowledge on engagement in healthcare decision making with a focus on older patients and their caregivers, promote the idea of patients and carers as equal partners, and supported the need of a discussion between them about needs and expectations [42]. Family meetings arranged by the hospital, have over time been found to be a robust format in that respect [20, 43]. Relatives have reported as most satisfying the information conveyed in family meetings and the subsequent discussions with the professionals [43]. Our study supports such forums of conversation between professionals, patient and family.

### Limitations

Data saturation has been discussed as a nebulous concept, but a presumptive ideal for which to strive [34].The study was exploratory and the sample size limited. However, it included participants of a wide range of ages and with different relations to the patient, allowing us to explore the data from a generational perspective. We cannot claim to have achieved maximum variation within this limited sample. Considering that most patients in the wards were 70 years and over, the potential participants within the group of younger adults was limited. The younger adult group in this study consisted of only one participant, which is a limitation. Furthermore, the middle-aged relatives stood out by having many siblings and other family members to help, which is not always the case. The older caregivers included in the study did not manage the care responsibility assigned to them, which is not always the case. It would also have been preferable with more participants from both genders.

The hospitals’ services from which we recruited participants, experienced time constraints and tight fiscal management at the time of data collection (2013). This might have impacted on the nurses who recruited participants, and might have raised the possibility of selection bias. However, our analysis indicates that the relatives who agreed to participate provided balanced accounts of their experiences, comprising both negative and positive elements.

## Conclusions

Our findings suggest that experiences of influence were limited regardless of age. However, the results indicate that user participation among relatives decrease with age, while vulnerability due to not having influence seems to increase with age. The *problem* of patient choice most clearly manifested themselves among the older carers. This might indicate that the relatives’ age sets terms for opportunities to participate.
